# Factors associated with the survival outcomes of patients with untreated hepatocellular carcinoma: An analysis of nationwide data

**DOI:** 10.3389/fonc.2023.1142661

**Published:** 2023-03-22

**Authors:** Min Jung Kwon, Soy Chang, Ji Hoon Kim, Ji Won Han, Jeong Won Jang, Jong Young Choi, Seung Kew Yoon, Pil Soo Sung

**Affiliations:** ^1^ Division of Gastroenterology and Hepatology, Department of Internal Medicine, College of Medicine, The Catholic University of Korea, Seoul, Republic of Korea; ^2^ The Catholic University Liver Research Center, College of Medicine, The Catholic University of Korea, Seoul, Republic of Korea

**Keywords:** hepatocellular carcinoma, tumor stage, MELD score, fetoprotein (AFP), survival & prognosis

## Abstract

**Introduction:**

In this study, we examined the natural course of untreated hepatocellular carcinoma (HCC) and identified predictors of survival in an area where hepatitis B is the predominant cause of HCC.

**Methods:**

We identified 1,045 patients with HCC who did not receive HCC treatment and were registered in the Korean Primary Liver Cancer Registry between 2008 and 2014, and were followed-up up to December 2018. Thereafter, we analyzed the clinical characteristics of patients who survived for <12 or ≥12 months. A Cox proportional regression model was used to identify the variables associated with patient survival.

**Results and discussion:**

The mean age of the untreated patients at HCC diagnosis was 59.6 years, and 52.1% of patients had hepatitis B. Most untreated patients (94.2%) died during the observation period. The median survival times for each Barcelona Clinic Liver Cancer (BCLC) stage were as follows: 31.0 months for stage 0/A (n = 123), 10.0 months for stage B (n = 96), 3.0 months for stage C (n = 599), and 1.0 month for stage D (n = 227). Multivariate Cox regression analysis demonstrated that BCLC stage D (hazard ratio, 4.282; P < 0.001), model for end-stage liver disease (MELD) score ≥10 (HR, 1.484; P < 0.001), and serum alpha-fetoprotein (AFP) level ≥1,000 ng/mL (HR, 1.506; P < 0.001) were associated with poor survival outcomes in patients with untreated HCC. In untreated patients with HCC, advanced stage BCLC, serum AFP level ≥1,000 ng/mL, and MELD score ≥10 were significantly associated with overall survival.

## 1 Introduction

Primary liver cancer is the sixth most commonly diagnosed cancer and the third leading cause of cancer-related deaths worldwide (1). Hepatocellular carcinoma (HCC) accounts for 75–85% of primary liver cancers and is a major healthcare issue (1, 2). The average crude incidence rate of HCC in Korea over the past 10 years was 22.4/100,000 person-years (3). Among patients in Korea, hepatitis B virus (HBV) infection is the leading cause of HCC (65%), followed by hepatitis C virus (HCV) (10%), and other causes (25%) (3–5). Despite advances in the diagnosis and treatment of HCC, prognoses following tumor development remain poor, with 5-year survival rates of 33.6% in the Republic of Korea and 18.1% in other Asian countries (3). Recent advances in immune-based therapies have enabled survival in patients with advanced HCC (6, 7).

To date, a considerable number of patients with HCC have remained untreated. A recent Korean study has addressed this issue. According to the results of a National Health Insurance Service database study in Korea, 27.6% of patients with HCC were left untreated between 2008 and 2013. In this study, the risk of mortality was higher for untreated patients in every subgroup, and the fully-adjusted hazard ratio (HR) for all-cause mortality comparing untreated to treated patients was 3.11 (95% confidence interval, 3.04–3.18) (8). However, this study employed an administrative claims dataset that lacked data pertaining to important clinical variables, such as performance status, Barcelona Clinic Liver Cancer (BCLC) stage, and tumor markers (8). Further research regarding clinical variables is necessary to determine the natural course of untreated HCC.

In an analysis of 128 hospitals by the United States (US) Veterans Administration, 24% of patients with HCC were untreated, and the median overall survival (OS) time was 3.6 months. This study also identified a model for end-stage liver disease (MELD) scores and alpha-fetoprotein (AFP) levels as prognostic factors in untreated patients, independent of the BCLC stage (9). Similarly, an analysis of an Italian liver cancer database that utilized data from 21 medical institutions showed that 11.7% of untreated patients had a median OS of 9 months (10). This study showed that female patients and those diagnosed with ascites or multinodular HCC were more likely to have advanced untreated HCC (10). However, it should be noted that both studies were performed with a largely HCV-positive cohort.

In this nationwide, multicenter, retrospective cohort study, we aimed to determine the natural course of untreated HCC in South Korea where HBV is the primary cause of HCC, and to identify predictors of survival in patients with untreated HCC. Data were obtained from a large, representative national cancer registry database that was based on random patient sampling and contained clinically important variables, such as laboratory findings, BCLC stage, and performance status.

## 2 Methods

### 2.1 Patients and study design

We used the KPLCR database, which contains hospital-based data from a randomly selected representative subset of patients with newly diagnosed HCC in Korea. The KPLCR contains data from approximately 15% of patients who were newly diagnosed with primary liver cancer and registered in the KCCR, a nationwide cancer registry that includes more than 95% of all cancer cases in Korea. Patients in the KPLCR were randomly selected from the KCCR using a probability proportional to size method after stratification by region within each year (11–13).

**Figure 1:**
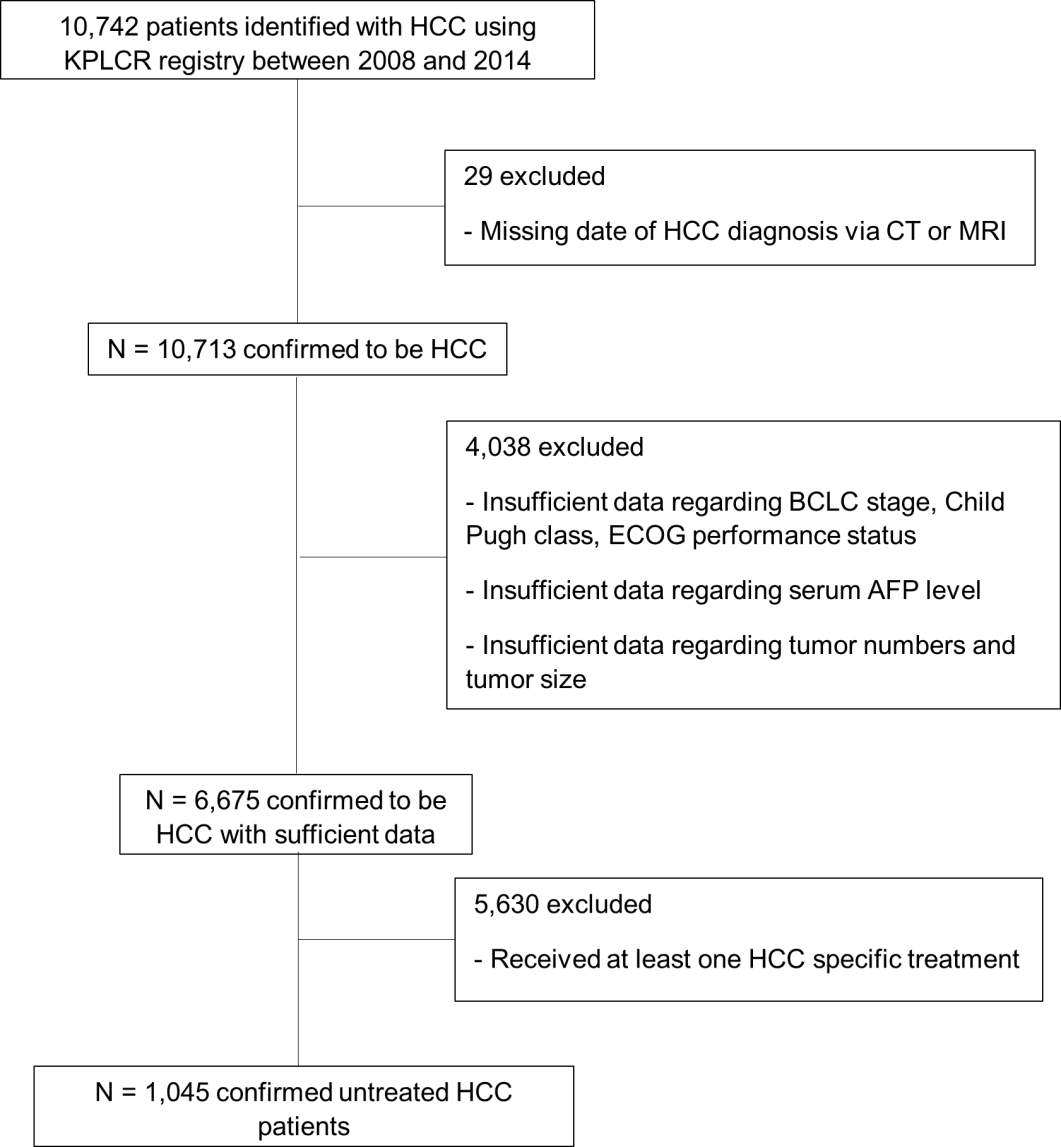
Flowchart showing the enrolment of study patients.

A total of 10,742 patients with HCC were identified using the KPLCR registry between 2008 and 2014. We excluded 29 patients with incomplete or missing data that were required for HCC diagnosis. We also excluded 4,038 patients with incomplete or missing data regarding major clinical parameters, such as BCLC stage and Child–Pugh class. Of the remaining patients, 5,630 had received at least one HCC-specific treatment and 1,045 had never received any HCC-specific treatment (**Figure 1**). We obtained data on the following characteristics of the 1,045 eligible patients from the KPLCR database: age, sex, BCLC stage, HCC etiology, Child–Pugh class, Eastern Cooperative Oncology Group (ECOG) performance status, initial serum AFP level, tumor count, tumor size, MELD score, portal vein invasion, encephalopathy, and ascites. HCC etiology was categorized as HBV, HCV, HBV and HCV co-infection, alcohol use, and others. HBV infection was confirmed by HBsAg positivity, HCV infection was confirmed by the presence of anti-HCV antibody (Ab) positivity, and HBV+HCV infection was confirmed by both HBsAg and anti-HCV Ab positivity. The initial serum AFP levels were dichotomized into ≥1000 ng/mL and <1000 ng/mL based on a previous study regarding factors predictive of “advanced” HCC (14–16). KPLCR is based on a national cancer registry program, in which all registered patients’ dates of death are recorded. Therefore, it is certain that patients without a date of death were in fact alive at the end of follow-up. Survival time was defined as the time (in months) from HCC diagnosis to death or end of follow-up (31 December 2018).

### 2.2 Statistical analysis

Data are presented as numbers with percentages for categorical variables and as means or medians (interquartile range) for continuous variables. Comparisons of categorical variables between groups were performed using Pearson’s chi-squared test. OS was calculated using the Kaplan–Meier method, and univariate analysis was performed using the log-rank test. Factors significantly associated with overall survival in the univariate analyses were considered potential input variables for the multivariate Cox proportional hazards regression analysis. We selected variables likely to be independent of one another concerning clinical interactions, followed by multivariate analysis to identify those that remained reliable predictors of survival in untreated patients with HCC. The results are presented as HRs with 95% confidence intervals. Statistical significance was set at *P* < 0.05. All statistical analyses were performed using SPSS software version 24.0 (SPSS Inc., Chicago, IL, USA).

## 3 Results

### 3.1 Baseline characteristics

Of the 6,675 patients with HCC, 1,045 did not receive any HCC-specific treatments, including resection, ablation, chemoembolization, chemotherapy, or liver transplantation; the remaining 5,630 received at least one HCC-specific treatment (**Figure 1**). The study cohort consisted of the 1,045 untreated patients with HCC. The mean age at HCC diagnosis was 59.55 years, and 80.2% of patients were male. In total, 123 (11.7%) patients had BCLC stage 0/A, 96 (9.2%) had stage B, 599 (57.3%) had stage C, and 227 (21.7%) had stage D disease at the time of diagnosis (**Table 1**). Compared with patients with HCC who had received treatment, the untreated group was older and had a more advanced BCLC stage, higher Child–Pugh class, higher MELD score, and poorer performance status at diagnosis (*P* < 0.001; **Table 1**). The untreated group also had higher serum AFP levels, higher tumor counts, larger tumors, and greater portal vein invasion at diagnosis (*P* < 0.001; **Table 1**). The most common etiology of HCC was HBV infection in both the treated (n = 3598, 63.9%) and untreated (n = 544, 52.1%; **Table 1**) groups.

**Table 1:** Characteristics of HCC patients who received and did not receive any treatment.

	Received no treatment, n = 1045 (%)	Received treatment, n = 5630 (%)	*P* value
Age, y			< .0001
<60 ≥60	457 (43.7) 588 (56.3)	2948 (52.4) 2682 (47.6)	
Sex			0.309
Male Female	838 (80.2) 207 (19.8)	4433 (78.7) 1197 (21.3)	
BCLC stage			
0/A (very early, early) B (intermediate) C (advanced) D (end stage)	123 (11.7) 96 (9.2) 599 (57.3) 227 (21.7)	2965 (52.6) 681 (12.1) 1860 (33.0) 124 (2.2)	< .0001
Etiology			< .0001
HBV HCV HBV+HCV Alcohol use Others	544 (52.1) 102 (9.8) 24 (2.3) 155 (14.8) 220 (21.1)	3598 (63.9) 608 (10.8) 71 (1.3) 600 (10.7) 753 (13.4)	
Child-Pugh class			< .0001
A B C	469 (44.9) 428 (41.0) 148 (14.2)	4803 (85.3) 748 (13.3) 79 (1.4)	
ECOG performance status			< .0001
0 1 2 3 4	550 (52.6) 265 (25.4) 119 (11.4) 65 (6.2) 46 (4.4)	4547 (80.8) 898 (16.0) 131 (2.3) 34 (0.6) 20 (0.4)	
Serum AFP level (ng/ml)			< .0001
<1000 ≥1000	610 (58.4) 435 (41.6)	4565 (81.1) 1065 (18.9)	
Tumor no.			< .0001
Single Multiple	439 (42.0) 606 (58.0)	3639 (64.6) 1991 (35.4)	
Tumor size			< .0001
< 5cm ≥ 5cm	279 (26.7) 766 (73.3)	3734 (66.3) 1896 (33.7)	
MELD score	(n = 984, missing = 61)	(n = 5471, missing = 159)	< .0001
<10 ≥10	401 (40.8) 583 (59.2)	3945 (72.1) 1526 (27.9)	
Portal vein invasion No Yes	517 (49.5) 528 (50.5)	4639 (82.4) 991 (17.6)	< .0001

Most untreated patients died within the observation period, with a median survival time of 3.0 months. The median survival times for each BCLC stage were as follows: 31.0 months for stage 0/A (n = 123), 10.0 months for stage B (n = 96), 3.0 months for stage C (n = 599), and 1.0 month for stage D (n = 227).

Considering that we extracted information from a nationwide database maintained by the government, the registry did not provide information regarding why some patients did not receive treatment for HCC. Therefore, we categorized the baseline characteristics of untreated patients as BCLC stage 0/A, B, and C/D, to explore reasons untreated patients did not receive the treatments, especially in the early stages (0/A and B) (**Supplementary Table 1**). The early-stage group (stage 0/A and B) included more patients over 60 years old, which may have influenced their decision on whether to receive treatment.

### 3.2 Factors affecting 12-month survival and OS

A chi-squared test comparing groups of patients who survived for <12 months and those who survived for ≥12 months showed that the following characteristics were associated with decreased 12-month survival rates (*P* < 0.001; **Table 2**): BCLC stage D disease classification, Child–Pugh class C, MELD score ≥10, serum AFP level ≥1000 ng/mL, poor performance status, moderate-to-severe ascites, multiple tumors, large tumor size (≥5 cm), and portal vein invasion.

**Table 2:** Potential determinants of survival among 1045 patients with untreated HCC.

	Total, N=1045	Survival <12 months, n = 810 (%)	Survival ≥12 months, n = 235 (%)	Mean survival months	*P* value
Age, y <60 ≥60	457 588	368 (80.5) 442 (75.2)	89 (19.5) 146 (24.8)	12.77 12.38	0.047
BCLC stage					< .0001
0/A (very early, early) B (intermediate) C (advanced) D (end stage)	123 96 599 227	35 (28.5) 50 (52.1) 505 (84.3) 220 (96.9)	88 (71.5) 46 (47.9) 94 (15.7) 7 (3.1)	41.33 22.08 8.87 2.64	
Etiology					0.001
HBV HCV HBV+HCV Alcohol use Others	544 102 24 155 220	446 (82.0) 70 (68.6) 18 (75.0) 106 (68.4) 170 (77.3)	98 (18.0) 32 (31.4) 6 (25.0) 49 (31.6) 50 (22.7)	11.57 12.74 10.42 16.5 12.37	
Child-Pugh class					< .0001
A B C	469 428 148	291 (62.0) 376 (87.9) 143 (96.6)	178 (38.0) 52 (12.1) 5 (3.4)	20.53 7.26 2.6	
MELD score (n = 984, missing = 61) <10 ≥10	401 583	261 (65.1) 513 (88.0)	140 (34.9) 70 (12.0)	18.42 7.4	< .0001
Serum AFP level (ng/ml) <1000 ≥1000	610 435	417 (68.4) 393 (90.3)	193 (31.6) 42 (9.7)	17.25 5.97	< .0001
ECOG performance status					< .0001
0 1 2 3 4	550 265 119 65 46	374 (68.0) 220 (83.0) 107 (89.9) 64 (98.5) 45 (97.8)	176 (32.0) 45 (17.0) 12 (10.1) 1 (1.5) 1 (2.2)	17.31 9.49 6.92 2.57 1.98	
Encephalopathy (n = 1042, missing = 3)					0.015
None Mild (confusion) Severe (Stupor or coma)	1006 27 9	773 (76.8) 26 (96.3) 9 (100.0)	233 (23.2) 1 (3.7) 0 (0.0)	12.85 4.41 1.44	
Ascites (n = 1037, missing = 8)					< .0001
None Mild Moderate to severe	523 297 217	339 (64.8) 257 (86.5) 208 (95.9)	184 (35.2) 40 (13.5) 9 (4.1)	19.64 7.04 3.28	
Tumor no.					< .0001
Single Multiple	439 606	297 (67.7) 513 (84.7)	142 (32.3) 93 (15.3)	18.48 8.26	
Tumor size					< .0001
< 5cm ≥ 5cm	279 766	148 (53.0) 662 (86.4)	131 (47.0) 104 (13.6)	15.84 6.07	
Portal vein invasion No Yes	517 528	315 (61.0) 495 (94.0)	202 (39.0) 33 (6.0)	21.13 4.16	< .0001

### 3.3 Univariate analysis for predictors of OS in untreated patients with HCC

**Figure 2:**
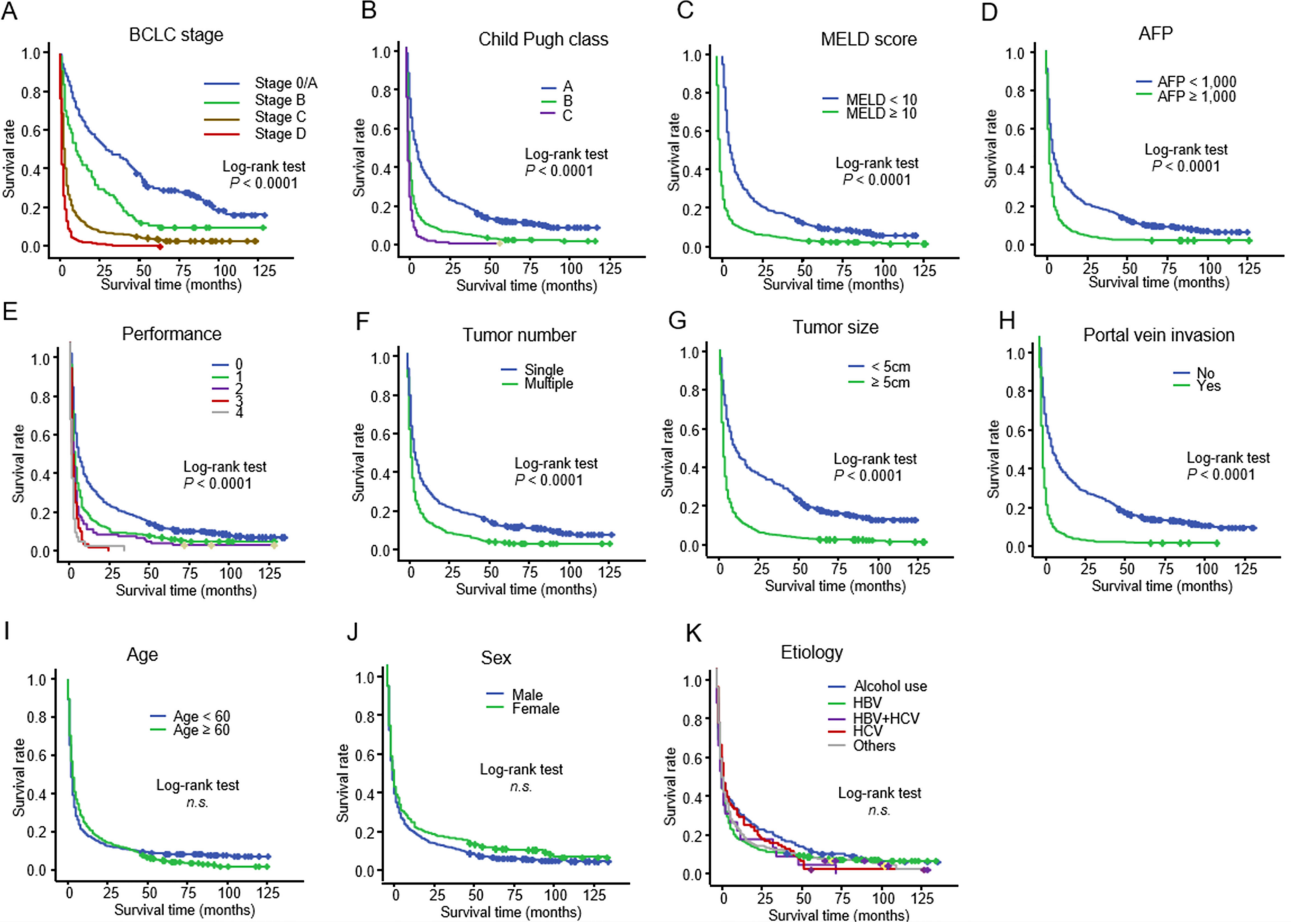
Kaplan–Meier curve showing the overall survival in 1,045 patients with untreated HCC stratified according to **(A)** BCLC stage: **(B)** Child–Pugh class: **(C)** MELD score: **(D)** serum AFP level: **(E)** ECOG performance score: **(F)** tumor number: **(G)** tumor size: **(H)** portal vein invasion: **(I)** age **(J)** sex: and **(K)** etiology HCC, hepatocellular carcinoma; BCLC, Barcelona Clinic Liver Cancer; MELD, model for end-stage liver disease; AFP, alpha-fetoprotein; ECOG, Eastern Cooperative Oncology Group.

According to the log-rank test, more advanced BCLC stage (A), more advanced Child–Pugh class (B), higher MELD score (C), higher serum AFP level (D), worse ECOG performance status (E), presence of ascites, history of encephalopathy, greater number of tumors (F), larger tumor size (G), and portal vein invasion (H) were reliable predictors of unfavorable OS (*P* < 0.001). However, the age (I), sex (J), and etiology (K) of HCC were not significantly associated with OS in patients with untreated HCC (**Figure 2**).

### 3.4 Multivariate analysis for predictors of OS in untreated patients with HCC

Two different multivariate analysis models were constructed based on a combination of factors that reliably predicted OS in the univariate analysis. Multivariate analysis model 1 (variables: BCLC stage, MELD score, and serum AFP) showed that BCLC stage D (HR, 4.282; *P* < 0.001), MELD score ≥10 (HR, 1.484; *P* < 0.001), and serum AFP level ≥1,000 ng/mL (HR, 1.506; *P* < 0.001) were associated with worse survival outcomes in untreated patients with HCC (**Table 3**). Multivariate analysis model 2 (variables: BCLC stage, serum AFP, and age) showed that BCLC stage D (HR = 5.155, *P* < 0.001) and serum AFP level ≥ 1,000 ng/mL (HR, 1.532; *P* < 0.001) were associated with worse survival outcomes in untreated patients with HCC (**Table 3**). Additionally, because of the increasing importance and correlation between metabolic syndrome and HCC, we also calculated hazard ratios of body mass index (BMI) and diabetes mellitus as variables associated with the prognosis of patients with untreated HCC. A BMI ≥ 25 kg/m^2^ was also associated with better prognosis (**Table 4**).

**Table 3:** Multivariate Cox Proportional Hazards Regression Models of Factors Associated with Mortality in 1045 Patients With Untreated HCC.

#1	Overall mortality	
Variable	HR (95% CI)	*P* value
BCLC stage 0/A B C D	Ref. 1.428 (1.050-1.942) 2.561 (2.026-3.238) 4.282 (3.272-5.604)	0.023 < .0001 < .0001
MELD score <10 ≥10	Ref. 1.484 (1.289-1.708)	< .0001
Serum AFP level (ng/ml) <1000 ≥1000	Ref. 1.506 (1.316-1.723)	< .0001
#2	Overall mortality	
Variable	HR (95% CI)	*P* value
BCLC stage 0/A B C D	Ref. 1.429 (1.051-1.944) 2.691 (2.130-3.399) 5.155 (3.961-6.711)	0.023 < .0001 < .0001
Serum AFP level (ng/ml) <1000 ≥1000	Ref. 1.532 (1.338-1.754)	< .0001
Age, y <60 ≥60	Ref. 1.126 (0.987-1.284)	0.078

**Table 4:** Multivariate cox proportional hazards regression models of factors associated with mortality in 806 patients with untreated HCC with information on BMI and diabetes.

#1	Overall mortality	
Variable	HR (95% CI)	*P* value
BCLC stage 0/A B C D	Ref. 1.293 (0.917-1.824) 2.441 (1.879-3.172) 3.878 (2.861-5.257)	0.142 < .0001 < .0001
MELD score <10 ≥10	Ref. 1.538 (1.315-1.799)	< .0001
Serum AFP level (ng/ml) <1000 ≥1000	Ref. 1.453 (1.249-1.690)	< .0001
BMI (kg/m^2^)		
<25	Ref.	
≥25	0.750 (0.636-0.886)	0.001
Diabetes		
No	Ref.	
Yes	0.972 (0.826-1.143)	0.731
#2	Overall mortality	
Variable	HR (95% CI)	*P* value
BCLC stage 0/A B C D	Ref. 1.322 (0.938-1.865) 2.566 (1.975-3.334) 4.789 (3.548-6.464)	0.111 < .0001 < .0001
Serum AFP level (ng/ml) <1000 ≥1000	Ref. 1.498 (1.287-1.743)	< .0001
Age, y <60 ≥60	Ref. 1.126 (0.971-1.307)	0.117
BMI (kg/m^2^)		
<25	Ref.	
≥25	0.802 (0.678-0.949)	0.010
Diabetes		
No	Ref.	
Yes	0.964 (0.818-1.136)	0.660

## 4 Discussion

Investigating the natural course of untreated HCC is essential to determine the factors that affect prognosis and the role of HCC screening with respect to lead-time bias (17). To our knowledge, this is the largest study to use a nationwide cohort of more than 1,000 untreated patients with HCC with detailed clinical information. Multivariate Cox regression analysis showed that advanced stage BCLC, serum AFP level, MELD score, and lower BMI were significantly associated with poor survival outcomes in untreated patients with HCC.

Previously, a meta-analysis reviewed 68 articles regarding prognostic factors of untreated patients with HCC and found that ECOG performance score, Child–Pugh B-C classes, presence of portal vein thrombosis, and presence of ascites were associated with poor survival in intermediate/advanced BCLC stages. However, in many of the studies included in the meta-analysis, the causes of HCC were missing; HCV status was missing in 11 of the 30 randomized controlled trial (RCTs) reviewed, HBV status was missing in six RCTs, and the proportion of alcohol consumption was not reported in 13 RCTs (18). Another study conducted in the US between 2004 and 2011 (n = 518) demonstrated that in untreated patients with HCC, advanced BCLC stage (A vs. D), MELD score (10–19 vs. <10, ≥20 vs. <10), and AFP level (≥1000 vs. <10 ng/mL) were predictive of 12-month mortality (17). In this study, a high percentage of patients had HCV infection, which is the major cause of HCC in the US.

In our study, a large proportion of patients had HBV infection, which is the major cause of HCC in Korea. As mentioned above, HBV is the leading cause of HCC (65%), followed by HCV (10%) and other causes (25%) in Korea (3, 4, 19). This aspect is notable since the study mentioned above was conducted in US veterans between 2004 and 2011, and consisted of a population in which a majority (60.6%) of the HCC study population had HCV infection (17). The median OS in their study was 3.6 months, whereas the median OS observed in this study was 3.0 months (17). Moreover, a study by Sinn et al. suggested differences in the clinical characteristics of HCC according to etiology including HBV-driven HCCs and HCV-driven HCCs. According to the study, the median age of diagnosis of HCC was higher in HCV-driven HCCs and the tumor size larger; furthermore, the presence of portal vein invasion was more frequent in HBV-driven HCCs than in HCV-driven HCCs (20). Unfortunately, because the KCCR is an anonymous nation-wide database, the data we extracted from the KPLCR was limited by several drawbacks, including the antiviral treatment history of patients with HBV and HCV, and the period of alcohol abstinence etiology.

Several factors may explain why the untreated patients with HCC in our study chose not to be treated. First, untreated patients with HCC are older and thus may have been less prone to aggressive treatment. Second, compared to treated patients with HCC whose percentage of HBV infection is 63.5%, the proportion of HBV infection is of a smaller degree (53.5%), and the cause of carcinogenesis is more concentrated on alcoholism and others, such as non-alcoholic steatohepatitis (NASH) or autoimmune hepatitis. This may also be because compared with younger patients, older patients are more prone to NASH-induced liver cirrhosis. Moreover, patients with alcoholic liver diseases are less compliant with treatment and surveillance than non-alcoholics, and alcoholics’ socioeconomic status tends to be on the downside, and they tend to be diagnosed with progressive HCC when it is too late for treatment. Moreover, in South Korea, there is a single-payer system and the National Health Insurance Service covers the entire population; hence, the treatment rates are higher than those in other nations. However, even with the single-payer system in place, treatment availability has been shown to vary according to income. These factors may explain why our pool of patients chose not to be treated but considering our retrospective characteristic of our study, it in an inevitable limitation. In South Korea, a nationwide cancer screening program has been implemented and credited with enhancing the outcomes of patients with HCC over the last two decades (21, 22). This program has shown that HBV-induced HCC is highly prevalent, yet compliance with the screening program has been suboptimal (23). Targeted screening programs for high-risk cohorts could increase the treatment rates and enhance the outlook of individuals with HCC.

This study had some limitations. As data were gathered retrospectively, the HCC stages and some prognostic covariates may have been misclassified. We also do not know whether untreated patients received any etiology-specific treatment (e.g., antiviral therapy), which may have confounded the patients’ liver function and overall survival. Our study is also biased towards the Korean population and further multinational studies will be needed, considering the differences that may exist, especially in the etiological origins of HCC.

## 5 Conclusion

For physicians treating patients with HCC, understanding the natural course of the disease is crucial. Using data from the Korean Primary Liver Cancer Registry of untreated patients with HCC from 2008 to 2014, we investigated those factors associated with prognosis. Overall, our study used a nationwide HCC registry to demonstrate that advanced BCLC stage, serum AFP level ≥1,000 ng/mL,MELD score ≥10, and higher BMI (≥25 kg/m^2^) were significantly associated with overall survival in untreated patients with HCC. Further multinational studies will reveal the natural history of HCC in populations where HBV is not the main cause of HCC.

## Data availability statement

Publicly available datasets were analyzed in this study. This data can be found here: Korean Primary Liver Cancer Registry.

## Ethics statement

The studies involving human participants were reviewed and approved by Institutional Review Board of St. Mary’s Hospital in Seoul. Written informed consent for participation was not required for this study in accordance with the national legislation and the institutional requirements.

## Author contributions

JK, MK, and SC contributed in the study concept, study design, interpretation of data, writing up of the first draft of the paper, critical revision of the manuscript for important intellectual content. JW and JJ contributed in study design, data analysis, interpretation of data, critical revision of the manuscript for important intellectual content. JC and SY contributed in the study design, data analysis, interpretation of data, critical revision of the manuscript for important intellectual content. PS conceived the idea for this study and participated in the study concept, study design, interpretation of data, critical revision of the manuscript for important intellectual content. All authors contributed to the article and approved the submitted version.

## References

[1] Sung H Ferlay J Siegel RL Laversanne M Soerjomataram I Jemal A. Global cancer statistics 2020: GLOBOCAN estimates of incidence and mortality worldwide for 36 cancers in 185 countries. CA Cancer J Clin (2021) 71:209–49. doi: 10.3322/caac.21660 33538338

[2] Reig M Forner A Rimola J Ferrer-Fabrega J Burrel M Garcia-Criado A. BCLC strategy for prognosis prediction and treatment recommendation: The 2022 update. J Hepatol (2022) 76:681–93. doi: 10.1016/j.jhep.2021.11.018 PMC8866082 34801630

[3] Chon YE Jeong SW Jun DW. Hepatocellular carcinoma statistics in South Korea. Clin Mol Hepatol (2021) 27:512–4. doi: 10.3350/cmh.2021.0171 PMC8273634 34153973

[4] Torimura T Iwamoto H. Optimizing the management of intermediate-stage hepatocellular carcinoma: Current trends and prospects. Clin Mol Hepatol (2021) 27:236–45. doi: 10.3350/cmh.2020.0204 PMC8046626 33317248

[5] Sung PS. Crosstalk between tumor-associated macrophages and neighboring cells in hepatocellular carcinoma. Clin Mol Hepatol (2022) 28:333–50. doi: 10.3350/cmh.2021.0308 PMC9293612 34665953

[6] Baretti M Kim AK Anders RA. Expanding the immunotherapy roadmap for hepatocellular carcinoma. Cancer Cell (2022) 40:252–4. doi: 10.1016/j.ccell.2022.02.017 PMC9844534 35290785

[7] Trifylli EM Koustas E Papadopoulos N Sarantis P Aloizos G Damaskos C. An insight into the novel immunotherapy and targeted therapeutic strategies for hepatocellular carcinoma and cholangiocarcinoma. Life (Basel) (2022) 12:665. doi: 10.3390/life12050665 35629333 PMC9146702

[8] Kim YA Kang D Moon H Sinn D Kang M Woo SM. Survival in untreated hepatocellular carcinoma: A national cohort study. PloS One (2021) 16:e0246143. doi: 10.1371/journal.pone.0246143 33539397 PMC7861368

[9] Serper M Taddei TH Mehta R D'Addeo K Dai F Aytaman A. Association of provider specialty and multidisciplinary care with hepatocellular carcinoma treatment and mortality. Gastroenterology (2017) 152:1954–64. doi: 10.1053/j.gastro.2017.02.040 PMC5664153 28283421

[10] Giannini EG Farinati F Ciccarese F Pecorelli A Rapaccini GL Di Marco M. Prognosis of untreated hepatocellular carcinoma. Hepatology (2015) 61:184–90. doi: 10.1002/hep.27443 25234419

[11] Lee JS Cho IR Lee HW Jeon MY Lim TS Baatarkhuu O. Conditional survival estimates improve over time for patients with hepatocellular carcinoma: An analysis for nationwide Korea cancer registry database. Cancer Res Treat (2019) 51:1347–56. doi: 10.4143/crt.2018.477 PMC6790830 30744320

[12] Yoon JS Lee HA Kim HY Sinn DH Lee DH Hong SK. Hepatocellular carcinoma in Korea: An analysis of the 2015 Korean nationwide cancer registry. J Liver Cancer (2021) 21:58–70. doi: 10.17998/jlc.21.1.58 PMC10035724 37384267

[13] Chon YE Lee HA Yoon JS Park JY Kim BH Lee IJ. Hepatocellular carcinoma in Korea between 2012 and 2014: An analysis of data from the Korean nationwide cancer registry. J Liver Cancer (2020) 20:135–47. doi: 10.17998/jlc.20.2.135 PMC10035678 37384317

[14] Lee J Han JW Sung PS Lee SK Yang H Nam HC. Comparative analysis of lenvatinib and hepatic arterial infusion chemotherapy in unresectable hepatocellular carcinoma: A multi-center, propensity score study. J Clin Med (2021) 10:4045. doi: 10.3390/jcm10184045 34575160 PMC8464794

[15] Sung PS Jang JW Lee J Lee SK Lee HL Yang H. Real-world outcomes of nivolumab in patients with unresectable hepatocellular carcinoma in an endemic area of hepatitis b virus infection. Front Oncol (2020) 10:1043. doi: 10.3389/fonc.2020.01043 32695681 PMC7338665

[16] Sung PS Choi MH Yang H Lee SK Chun HJ Jang JW. Diffusion-weighted magnetic resonance imaging in hepatocellular carcinoma as a predictor of a response to cisplatin-based hepatic arterial infusion chemotherapy. Front Oncol (2020) 10:600233. doi: 10.3389/fonc.2020.600233 33330098 PMC7711158

[17] Khalaf N Ying J Mittal S Temple S Kanwal F Davila J. Natural history of untreated hepatocellular carcinoma in a US cohort and the role of cancer surveillance. Clin Gastroenterol Hepatol (2017) 15:273–81.e1. doi: 10.1016/j.cgh.2016.07.033 27521507

[18] Cabibbo G Enea M Attanasio M Bruix J Craxi A Camma C. A meta-analysis of survival rates of untreated patients in randomized clinical trials of hepatocellular carcinoma. Hepatology (2010) 51:1274–83. doi: 10.1002/hep.23485 20112254

[19] Sung PS Park DJ Roh PR Mun KD Cho SW Lee GW. Intrahepatic inflammatory IgA(+)PD-L1(high) monocytes in hepatocellular carcinoma development and immunotherapy. J Immunother Cancer (2022) 10:e003618. doi: 10.1136/jitc-2021-003618 35577505 PMC9114848

[20] Sinn DH Gwak GY Cho J Paik SW Yoo BC. Comparison of clinical manifestations and outcomes between hepatitis b virus- and hepatitis c virus-related hepatocellular carcinoma: Analysis of a nationwide cohort. PloS One (2014) 9:e112184. doi: 10.1371/journal.pone.0112184 25372403 PMC4221592

[21] Yim SY Seo YS Jung CH Kim TH Lee JM Kim ES. The management and prognosis of patients with hepatocellular carcinoma: What has changed in 20 years? Liver Int (2016) 36:445–53. doi: 10.1111/liv.12960 26352789

[22] Im S Jang ES Lee JH Lee CS Kim BH Chung JW. Surveillance rate and its impact on survival of hepatocellular carcinoma patients in south Korea: A cohort study. Cancer Res Treat (2019) 51:1357–69. doi: 10.4143/crt.2018.430 PMC6790861 30744319

[23] The Korean Association for the Study of the Liver (KASL). KSL clinical practice guidelines for management of chronic hepatitis B. Clin Mol Hepatol (2022) 28:276–331. doi: 10.3350/cmh.2022.0084 35430783 PMC9013624

