# Supplementary material for: Factors associated with the survival outcomes of patients with untreated hepatocellular carcinoma: An analysis of nationwide data
Source: Front Oncol. 2023 Mar 22;13:1142661. doi: 10.3389/fonc.2023.1142661 (PMC10073541; doi:10.3389/fonc.2023.1142661)
Supplement: Supplementary file 1 [file Table_1.pdf]

Supplementary Material

Factors associated with the survival in patients with untreated hepatocellular carcinoma: analysis of nationwide data

Min Jung Kwon[1†], Soy Chang[1†], Ji Hoon Kim[1†], Ji Won Han[1,2], Jeong Won Jang[1,2], Jong Young Choi[1,2], Seung Kew Yoon[1,2], Pil Soo Sung[1,2]

*** Correspondence:**

Pil Soo Sung, M.D., Ph.D.

Division of Gastroenterology and Hepatology, Department of Internal Medicine, College of Medicine, Seoul St. Mary's Hospital, The Catholic University of Korea, Seoul 06591, Republic of Korea. E-mail: pssung@catholic.ac.kr

Supplementary Table 1. Baseline characteristics of untreated patients with HCC according to BCLC stage

	BCLC stage 0/A n = 123 (%)	BCLC stage B n = 96 (%)	BCLC stage C/D n = 826 (%)
Age, y			
<60	36 (29.3)	29 (30.2)	392 (47.5)
≥60	87 (70.7)	67 (69.8)	434 (52.5)
Sex			
Male	88 (71.5)	76 (79.2)	674 (81.6)
Female	35 (28.5)	20 (20.8)	152 (18.4)
Etiology			
HBV	50 (40.7)	37 (38.5)	457 (55.3)
HCV	15 (12.2)	16 (16.7)	71 (8.6)
HBV+HCV	3 (2.4)	1 (1.0)	20 (2.4)
Alcohol use	24 (19.5)	15 (15.6)	116 (14.0)
Others	31 (25.2)	27 (28.1)	162 (19.6)
ECOG performance status			
0	123 (100)	96 (100)	331 (40.1)
1	0 (0)	0 (0)	265 (32.1)
2	0 (0)	0 (0)	119 (14.4)
3	0 (0)	0 (0)	65 (7.9)
4	0 (0)	0 (0)	46 (5.6)
Child-Pugh class			
A	91 (74.0)	71 (74.0)	307 (37.2)
B	32 (26.0)	25 (26.0)	371 (44.9)
C	0 (0)	0 (0)	148 (17.9)
Diabetes mellitus	33 (29.2)	24 (25.8)	215 (26.7)
Hypertension	45 (40.2)	35 (37.6)	263 (32.7)
Smoker	39 (34.5)	42 (45.2)	402 (49.8)

** Data concerning the following were missing

Concerning DM:

- 0/A: 10 patients were missing information
- B: 3 patients were missing information
- C/D: 22 patients were missing information

Concerning smoking:

- 0/A: 10 patients were missing information
- B: 3 patients were missing information
- C/D: 18 patients were missing information

Concerning hypertension:

- 0/A: 11 patients were missing information
- B: 3 patients were missing information
- C/D: 21 patients were missing information

Statistical analysis was conducted excluding patients with missing information.
